# Identification of COVID-19 Infection-Related Human Genes Based on a Random Walk Model in a Virus–Human Protein Interaction Network

**DOI:** 10.1155/2020/4256301

**Published:** 2020-07-08

**Authors:** YuHang Zhang, Tao Zeng, Lei Chen, ShiJian Ding, Tao Huang, Yu-Dong Cai

**Affiliations:** ^1^School of Life Sciences, Shanghai University, Shanghai 200444, China; ^2^Shanghai Institute of Nutrition and Health, Shanghai Institutes for Biological Sciences, Chinese Academy of Sciences, Shanghai 200031, China; ^3^Key Laboratory of Systems Biology, Institute of Biochemistry and Cell Biology, Chinese Academy of Sciences, Shanghai 200031, China; ^4^College of Information Engineering, Shanghai Maritime University, Shanghai 201306, China

## Abstract

Coronaviruses are specific crown-shaped viruses that were first identified in the 1960s, and three typical examples of the most recent coronavirus disease outbreaks include severe acute respiratory syndrome (SARS), Middle East respiratory syndrome (MERS), and COVID-19. Particularly, COVID-19 is currently causing a worldwide pandemic, threatening the health of human beings globally. The identification of viral pathogenic mechanisms is important for further developing effective drugs and targeted clinical treatment methods. The delayed revelation of viral infectious mechanisms is currently one of the technical obstacles in the prevention and treatment of infectious diseases. In this study, we proposed a random walk model to identify the potential pathological mechanisms of COVID-19 on a virus–human protein interaction network, and we effectively identified a group of proteins that have already been determined to be potentially important for COVID-19 infection and for similar SARS infections, which help further developing drugs and targeted therapeutic methods against COVID-19. Moreover, we constructed a standard computational workflow for predicting the pathological biomarkers and related pharmacological targets of infectious diseases.

## 1. Introduction

Coronaviruses are specific crown-shaped viruses that were first identified in the 1960s [1, 2]. In the 1960s, they were first identified as pathogens for zoonotic diseases without a direct and clear origin trace [2]. They are highly transmissible viruses that can be spread via droplets and skin contact [3, 4]. Most coronaviruses are widely spread around the world. They cause simple and mild symptoms that are the same as cold and mild flu symptoms. However, specific coronavirus subtypes cause severe and even deadly symptoms, inducing large-scale pandemics regionally or worldwide. Three typical examples of the most recent coronavirus disease outbreaks include severe acute respiratory syndrome (SARS) [5, 6], Middle East respiratory syndrome (MERS) [7, 8], and COVID-19 [9, 10].

In 2003, the SARS outbreak occurred; it spread to more than 20 countries and regions in the Eurasian continent and resulted in the deaths of 774 people [5, 6]. The typical symptoms of SARS are high fevers in the early stage and severe inflammations in lung tissues after 2–7 days of quick progression [11]. The large-scale SARS pandemic ended in less than half a year as a result of the effective epidemiological control exerted by the government [11]. After a decade, MERS, another deadly and highly transmissible coronavirus, emerged in 2012. MERS has more severe early cellular infection and invasion capacity than SARS [7]. It causes typical symptoms in less than 24 h, whereas SARS causes symptoms in 72 h. Therefore, treating and curing patients infected with MERS are more difficult than treating and curing patients with SARS [8]. At the end of 2019, COVID-19, a new coronavirus subtype, broke out. In accordance with current epidemiological and clinical data, COVID-19 has been confirmed to be a similar coronavirus subtype as MERS and SARS [3]. However, compared with the two other coronavirus subtypes, COVID-19 causes more complicated clinical symptoms [9] and has higher transmissible capacity [3] and faster mutational rates [9]. These characteristics make its prevention and treatment difficult. More than 150,000 people have been confirmed to be infected with COVID-19, which has caused 5735 deaths. COVID-19 is currently causing a worldwide pandemic, threatening the health of human beings globally [9].

The identification of viral pathogenic mechanisms is important for further developing effective drugs and targeted clinical treatment methods. The delayed revelation of viral infectious mechanisms is currently one of the technical obstacles in the prevention and treatment of infectious diseases. Although SARS and MERS have already been finally controlled by regional governments, the pathogenic mechanisms of these diseases have not been fully revealed [12, 13]. SARS was controlled and banished in 2003, but its detailed mechanisms were not finally determined until 2005 [14, 15]. Meanwhile, the pathogenic mechanisms of MERS have still not been fully identified. The investigation of the pathological mechanisms of virulent viral pathogens by using traditional methods remains challenging.

In this study, we proposed a computational method to identify the potential pathological mechanisms of COVID-19, the coronavirus subtype that is now spreading all over the world and causing the 2019–2020 coronavirus pandemic. By using our prediction model, which is based on a random walk algorithm on a virus–human protein interaction network, we effectively identified a group of proteins that have already been determined to be potentially important for COVID-19 infection and for similar SARS infections. Through our newly presented computational methods, we identified a group of potential biomarkers for further developing drugs and targeted therapeutic methods against COVID-19. Moreover, we constructed a standard computational workflow for predicting the pathological biomarkers and related pharmacological targets of infectious diseases.

## 2. Materials and Methods

### 2.1. Dataset Construction of Target Human Proteins

Similar to the construction strategy used in a previous work [16], protein–protein interactions (PPIs) between the virus and its host (human) can be used to determine the course of COVID-19 infection. Whether a human protein interacts with viral proteins can be determined on the basis of functional terms from the Gene Ontology (GO) database [17]. A human protein and COVID-19 protein that shared at least 1 GO term were assumed to interact with each other. The human protein was called the target human protein. Only GO terms at levels below 3 were considered to remove protein pairs sharing generic GO terms. For example, root GO terms (“GO: 0008150: biological process,” “GO: 0005575: cellular component,” and “GO:0003674: molecular function”), their children, and the children of their children terms were ignored in the following analysis. Through this approach, we constructed a dataset of target human proteins. All protein sequences of COVID-19 (Reference Genome MN908947) were downloaded from the NCBI protein database (http://www.ncbi.nlm.nih.gov/) in accordance with a preprinted article of Fast and Chen [18], and the sequences of the 11 COVID-19 proteins (orf1ab, S, orf3a, orf6, E, M, orf8, N, orf7b, orf7a, and orf10) are listed in Supporting Information S1.

### 2.2. PPI Data from STRING

Search Tool for the Retrieval of Interacting Genes (STRING) (http://string.embl.de/) is an online database resource. It compiles experimental and predicted PPIs with a confidence score. The PPIs in STRING are derived from several sources, such as (conserved) coexpression, automated text mining, genomic context predictions, high-throughput lab experiments, and previous knowledge in databases. Accordingly, they can widely measure the associations of proteins, which is a great advantage compared with PPIs reported in other databases, such as DIP (Database of Interaction Proteins) database [19] and BioGRID [20]. Thus, we collected a weighted PPI network *G* from STRING, in which the network nodes represent proteins and the network edges represent interactions between proteins with weights that indicate the significance of shared similar biological functions [21, 22]. Notably, the weight of each interaction edge was assigned with a weight, which was defined as the original confidence score reported in STRING. In this study, we analyzed the network wherein every two proteins in one interaction were in the target human protein dataset. The constructed network contained 19,247 nodes and 4,274,001 edges.

### 2.3. Random Walk with Restart Algorithm

The random walk with restart (RWR) algorithm, one of the typical network-based feature ranking algorithms [23, 24], can simulate a random walker that starts from one or several seed nodes and walks on a network.

RWR can update the weight (probability) vector of network nodes in an iterative manner in accordance with the following mathematical description: let *P*_*i*_ be the probability vector after the *i*^th^ updating procedure. The next new probability vector *P*_*i*+1_ will be updated depending on the previous vector *P*_*i*_ as

(1) $$\begin{array}{c} P_{i+1}=\left(1-\lambda \right)AP_{i}+\lambda P_{0}, \end{array}$$

where *A* is the column-wise normalized adjacency matrix of the given network, *λ* indicates the probability of the walker returning to the seed nodes, and *P*_0_ is an initial probability vector. When the probability vector becomes convergent, the RWR algorithm stops and outputs the final *P*_*i*+1_. Each element of the final *P*_*i*+1_ indicates the probability that the corresponding nodes are associated with the seed nodes.

In this work, 11,419 mapped candidate human proteins shared the same GO functions of COVID-19 were picked up as seed nodes in the RWR algorithm. Initialization vector *P*_0_ was constructed. It consisted of 19,247 elements, wherein the elements corresponding to the seed genes were set to 1/11419, the other elements were set to zero, and the probability of returning to seed node *λ* was set to 0.8 as suggested in some studies [25–29]. The algorithm termination rule was ‖*P*_*i*+1_ − *P*_*i*_‖_1_ < 10^−6^.

### 2.4. Permutation Test

Based on the RWR algorithm, each protein in the PPI network was assigned a probability, which can indicate the associations between it and seed nodes. However, this value was determined by not only the seed nodes but also the topological structure of the network. Some special nodes in the network may more easily receive high probabilities than others. To control the influence of such factor, a permutation test was designed. First, we randomly constructed 1000 node sets, each of which contained 11,419 nodes. Second, for each node set, it was picked up as the input of the RWR algorithm; accordingly, each node in the network received a lot of probabilities. Finally, a *P* value was computed for each node *n*, which was defined by

(2) $$\begin{array}{c} P\text{ value}\left(n\right)=\frac{m}{1000}, \end{array}$$

where *m* denoted the number of probabilities on the randomly produced node sets that were larger than the probability on the actual seed nodes. Clearly, a node with a low *P* value indicated that it was special for the seed nodes. Considering that 0.05 is a widely used threshold for statistical significance, we selected nodes with *P* values less than 0.05 and their corresponding proteins were picked up for detailed analysis.

## 3. Results and Discussion

**Figure 1:**
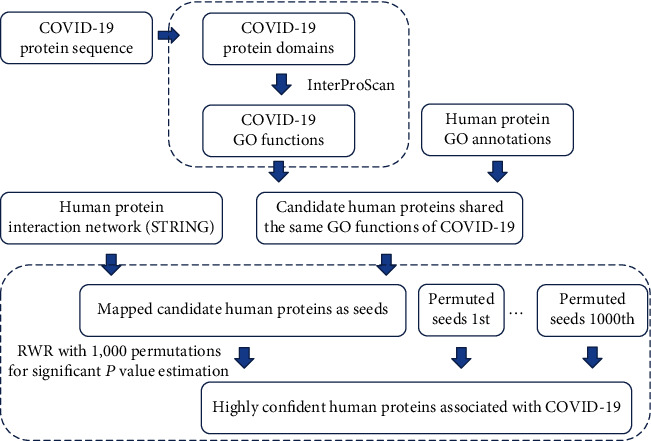
Analysis flow chart of the identification of COVID-19 infection-related human genes. First, the Gene Ontology (GO) functions of COVID-19 proteins are extracted. Second, human proteins sharing these GO functions are selected. Third, these human proteins are set as the input of the random walk with restart (RWR) algorithm, which is applied to the protein interaction network reported in STRING. Finally, the permutation test followed to further select human proteins with significant *P* values.

In this study, we designed a computation method to investigate the COVID-19 infection-related human genes. The entire procedures are shown in Figure 1.

### 3.1. Human Proteins Sharing Common GO Functions with COVID-19 Proteins

We downloaded the sequences of 11 COVID-19 proteins which are listed in Supplementary file 1. We predicted the domains and GO functions of these virus proteins based on their sequences using InterProScan (http://www.ebi.ac.uk/interpro/search/sequence/), and the InterPro results are given in Supplementary file 2. The 20 GO terms with level ≥ 3 were selected for the following analysis, as listed in Supplementary file 3. Next, we extracted the human proteins shared with the same 20 GO functions and these candidate human proteins are listed in Supplementary file 4. Then, we mapped them into the STRING network and finally obtained 11,419 proteins (Supplementary file 5).

### 3.2. Results of the RWR Algorithm and Permutation Test

After the above data preparation, we applied the RWR method with these 11,491 proteins as seeds on the STRING network and calculated the RWR probabilities of all proteins on the network. Meanwhile, we randomly selected the same number of seed proteins 1000 times and calculated all proteins' permuted RWR probabilities. By comparing the actual and 1000 permutated RWR probabilities, we estimated the *P* value of a protein being truly associated with COVID-19. Finally, we captured 486 highly confident human proteins associated with COVID-19 according to their RWR probabilities and permutation *P* values (<0.05), as listed in Supplementary file 6. We analyzed and discussed a few representative candidates as listed in Table 1 in reference to recent reports on their functions.

### 3.3. Analysis on Some Essential Human Proteins

On the basis of our newly presented computational method, we applied the RWR algorithm to identify potential proteins that might functionally interact with the infectious process of COVID-19, which causes the typical disease coronavirus disease-19. According to recent publications, such proteins may not be only functionally related to the infection process of COVID-19 but may also participate in the infectious process of SARS, another famous infectious disease of the respiratory system. The detailed analyses can be seen below.

The first protein is ubiquitin-like 4A (UBL4A, **ENSP00000358674**). This protein, which is one of the major functional components of the BAG6/BAT3 complex, has been widely reported to participate in the recognition and delivery of misfolded and hydrophobic patch-containing proteins to proteasomes for degradation [30, 31]. In 2017, this protein was confirmed to participate in the endoplasmic-reticulum-associated protein degradation (ERAD) pathway in the viral infection cycle [32]. In fact, the ERAD pathway has been reported to participate in the infection processes of various well-known viruses, e.g., the ERAD pathway has been confirmed to participate in the pathogenesis of the SARS coronavirus [33]. Given that the infectious mechanism of COVID-19 has also been confirmed to be similar to that of SARS, we can reasonably predict that the ERAD pathway and one of its specific components, UBL4A, contribute to the pathogenesis of COVID-19 infection [34]. Another predicted protein, ubiquitin-like 4b (UBL4B, **ENSP00000334044**), was identified by our newly presented computational models. UBL4B is also a component of the ERAD pathway and is therefore definitely functionally correlated with the pathogenesis of multiple coronaviruses, including SARS and COVID-19 [33, 34].

The next protein identified was uridine monophosphate synthetase (UMPS, **ENSP00000232607**), which contributes to the de novo pyrimidine biosynthetic pathway. As a biofunctional enzyme, this protein can help convert orotic acid into orotidine-5′-monophosphate and further convert orotidine-5′-monophosphate into uridine monophosphate [35, 36]. Uridine monophosphate, the terminal product of UMPS, has been widely reported to participate in coronaviral infection processes [37–39], especially RNA-polymerase-associated processes [38, 39]. In contrast to the infection processes of other coronaviruses, SARS infection exhibits abnormal uridine monophosphate regulation [38]. Although experimental evidence remains lacking, we can still reasonably speculate that UMPS is functionally correlated with the COVID-19 infectious process considering that uridine monophosphate and its regulator UMPS are essential for RNA polymerases in RNA viruses, such as COVID-19.

POTEF (**ENSP00000350052**) is the next predicted protein that potentially contributes to COVID-19 infection. Similar to UBL4A, POTEF is a specific protein that contributes to the regulation of protein binding [40, 41]. This protein has been identified as A26C1B in various infectious models. In 2010, it was found to participate in the infection of HIV in chimpanzees [42]. Furthermore, POTE, the family of POTEF, contributes to macrophage-mediated antiviral biological processes [43]. Considering that the pathogeneses of SARS and other coronavirus have been confirmed to interact with macrophages and related immune responses [44] and macrophage infiltration in the lungs has already been widely reported [45, 46], this protein may also participate in COVID-19 pathogenesis.

The next predicted protein is LOC101927789 (**ENSP00000310146**). This novel identified fusion pseudogene has been identified to be a reactor against chemical exposure and is functionally correlated with another effective protein, MALAT1 [47]. Recent publications confirm that MALAT1 is functionally related to the infectious processes of various viruses, including coronaviruses [48–50]. Although direct evidence showing that our predicted protein contributes to the infection of coronaviruses (SARS or COVID-19) remains lacking, we can reasonably speculate that MALAT1, together with our predicted fusion pseudogene, participates in some conserved biological or pathological processes of coronaviruses.

The final discussed protein is UBBP4 (**ENSP00000464265**), which has been widely reported to act as a pseudogene and is functionally correlated with psoriasis [51, 52]. Similar to UBL4A and UBL4B, UBBP4 can contribute to the regulation of the ERAD pathway [53] and therefore may be functionally correlated with COVID-19 infection. This relationship validates the efficacy and accuracy of our prediction.

## 4. Conclusions

All the predicted proteins were functionally confirmed to contribute to viral and coronaviral infection processes. Notably, many of the predicted proteins were functionally correlated with protein degradation and RNA metabolism, which are essential for viral infection, implying their potential functional relationships with COVID-19 infection. Our results will help design drugs and targeted therapy against COVID-19.

## Figures and Tables

**Table 1:** Representative candidate of COVID-19 infection-related human genes.

Ensembl ID	Probability	*P* value	Gene name
ENSP00000358674	7.86*E* − 05	<0.001	UBL4A
ENSP00000367869	7.65*E* − 05	<0.001	GNB1
ENSP00000232607	7.53*E* − 05	<0.001	UMPS
ENSP00000350052	7.20*E* − 05	0.010	POTEF
ENSP00000334044	6.61*E* − 05	<0.001	UBL4B
ENSP00000310146	6.55*E* − 05	<0.001	None
ENSP00000464265	6.55*E* − 05	<0.001	UBBP4
ENSP00000355865	6.17*E* − 05	<0.001	PARK2
ENSP00000340944	5.67*E* − 05	0.027	PTPN11
ENSP00000377751	5.65*E* − 05	0.034	SCOC

## Data Availability

No data were used to support this study.

## Supplementary Materials

Supplementary Materials Supplementary File 1: the sequences of COVID-19 proteins. Supplementary File 2: the predicted domains and GO functions of COVID-19 proteins by InterProScan. Supplementary File 3: the GO terms with level ≥ 3 of COVID-19 proteins. Supplementary File 4: the candidate human proteins shared the same GO functions of COVID-19. Supplementary File 5: the mapped COVID-19 candidate human proteins on STRING network. Supplementary File 6: the highly confident human proteins associated with infection of COVID-19.
